# Structural and Superconducting Proximity Effect of SnPb Bimetallic Nanoalloys

**DOI:** 10.3390/nano12234323

**Published:** 2022-12-05

**Authors:** Ashish Chhaganlal Gandhi, Krishtappa Manjunatha, Ting-Shan Chan, Sheng Yun Wu

**Affiliations:** 1 Department of Physics, National Dong Hwa University, Hualien 97401, Taiwan; 2 Department of Electrical Engineering, National Tsing Hua University, Hsinchu 30013, Taiwan; 3 National Synchrotron Radiation Research Center, Hsinchu 30076, Taiwan

**Keywords:** superconducting proximity effect, electron–phonon coupling, superconductor, SnPb bimetallic system

## Abstract

We report the superconducting properties between a conventional strong-coupled Pb and weak-coupled Sn superconductor. A series of Sn_r_Pb_1-r_ nanoalloys with various compositions r were synthesized, and their superconducting properties were measured using superconducting quantum interference devices (SQUIDs) magnetometer. Our results reveal a superconducting proximity effect (SPE) between immiscible Sn and Pb granules in the range of r = 0.2~0.9, as a weak superconducting coupling can be established with the coexistence of phonon hardening and increased Ginzburg–Landau coherence length. Furthermore, our results provide new insights into improving the study of the superconducting proximity effect introduced by Sn doping.

## 1. Introduction

The concept of the proximity effect between a superconductor (SC) and a standard metal (NM) induced superconductivity was developed in 1960 [1] At the interface of SC/NM, one can observe the breaking of the Cooper pair in the SC at the length scale of the coherence length (ξ) across the interface. The applying boundary condition at the interface, the pairing amplitude $F=\langle \Psi_{↑}\Psi_{↓}\rangle ,$ is suppressed at the surface of the SC and enhanced in NM. Recently, much attention has been paid to search for Majorana fermions (MFs) in condensed matter systems using a conventional *s*-wave SC-3D topological insulator (TI), where a proximity-induced state resembling a spinless superconductor is expected to occur [2]. Lu et al. investigated the conductance spectra of Sn-Bi_2_Se_3_ interface junctions down to 250 mK and in different magnetic fields [3]. As a result, a proximity-effect-induced chiral superconducting phase is found and formed at the interface between the superconducting Sn and the strong spin-orbit coupling material Bi_2_Se_3_.

Moreover, a Josephson current can be established over several microns in the lateral direction between two Pb- or Sn-electrodes on the Bi_2_Te_3_ surface, demonstrating that superconducting quantum interference devices can be constructed based on proximity-effect-induced superconductivity [4,5]. *The interplay of* BCS superconductivity and nontrivial band topology is expected to give rise to the search for *Majorana fermion* quasiparticles in condensed matter systems. Therefore, knowledge of the precise electron–phonon and superconducting coupling strength of Pb/Sn is essential in explaining the proximity effect.

In the last two decades, researchers have shown interest in the Pb/Sn system to study the superconducting proximity effect (SPE) [6,7,8]. The nanoscale system has attracted further interest to study the size effects on the superconducting to normal state transition, on the flux-line penetration in a spatially confined region, or on the phase-slip mechanism [6]. In bimetallic superconducting nanoalloys with a heterogeneous distribution of grains more minor than the ξ of the constituent elements, the superconducting proximity effect (SPE) can alter the superconducting properties. In this work, we report the observation of the SPE on the superconducting properties of Sn_r_Pb_1-r_ (0.01 ≤ r ≤ 0.99) bimetallic nanoalloys. Granular Sn can be randomly distributed within the Pb matrix during the alloying process. In this scenario, if the grain size is smaller than the superconducting characteristic length scales, then studying SPE can give more insight into the superconducting properties of immiscible bimetallic nanoalloys.

## 2. Methods

Sn_r_Pb_1-r_ (0.01 ≤ r ≤ 0.99) superconductors were prepared using a physical solid-state reaction. This Sn/Pb concentration tuning alters the crystal structure, thereby changing the superconducting properties. Surface morphological analysis and atomic percentage calculation of all the samples were performed by field-emission scanning electron microscopy (FE-SEM) using a JEOL JSM-6500F microscope (JEOL Ltd., Tokyo, Japan). Energy dispersive spectroscopy (EDS; Inca x-sight model 7557, Oxford Instruments, Abingdon, Oxfordshire, UK) was utilized to estimate the atomic percentages of the constituent elements. Energy-dispersive spectroscopy (EDS) is a valuable technique for estimating the samples’ atomic rates of constituent elements. To study the alloying effect of Sn and Pb on structural properties, synchrotron radiation X-ray diffraction (SR-XRD) of the nanoalloys was carried out using synchrotron radiation beamline BL-01C2 at the National Synchrotron Radiation Research Center (NSRRC) in Taiwan with an incident wavelength λ = 0.7749 Å.

## 3. Results

### 3.1. Elemental Analysis and EDS Mapping of SnPb

**Figure 1:**
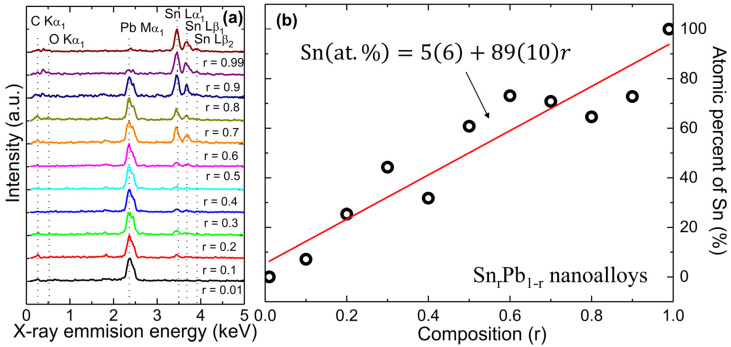
(**a**) Typical EDS elemental spectra reveal a series of peaks associated with Sn and Pb elements, verifying that the Sn_r_Pb_1-r_ bimetallic nanoalloys contain only Sn and Pb elements; (**b**) atomic % of Sn with respect to the initial composition of Sn_r_Pb_1-r_ bimetallic nanoalloys.

Figure 1a shows the typical EDS spectra of a series of Sn_r_Pb_1-r_ (0.01 ≤ r ≤ 0.99) bimetallic nanoalloys, which are shifted vertically for clarity. The series of Sn and Pb constituent elements observed in EDS spectra are assigned to Pb-Mα_1_, Sn-Lα, Sn-Lβ_1_, and Sn-Lβ_2_. The weak peak of C-Kα1 and O-Kα1 in the low energy regions originated from the carbon tape used for mounting the sample and surface oxygen, respectively. Figure S1a–k (Supporting Information) presents the EDS mapping with SEM images of Sn_r_Pb_1-r_ bimetallic nanoalloys, where red and green represent the atomic percent of Pb and Sn, respectively. Figure 1b shows the plot of atomic % of Sn concerning composition r, which increases linearly with a slope of 89 (10) atomic percentage with Sn composition (at. %/r). The observed slight discrepancy of at. % with initial composition r could be due to the inhomogeneous distribution of constituent elements. The phase diagram is very sensitive to preparation conditions, so slight changes in the temperature, pressure, or initial composition can change the weight percent of the constituent phases for each nanoalloy. The EDS mapping images of Sn_r_Pb_1-r_ nanoalloys show the distribution of segregated Sn (bright green color) and Pb (red color) elements with grain size varying from <*d*> = 121 (10)~46 (3) nm. The observed discrepancy of atomic % with initial composition is due to three-dimensional inhomogeneous distributions of segregated grains of Sn and Pb. In such a scenario magnetization, M(T) can be fitted with the London equation of granular sized <*d>,* which will be discussed in the magnetization section.

### 3.2. Crystal Structural and X-ray Refinement Analysis of SnPb

**Figure 2:**
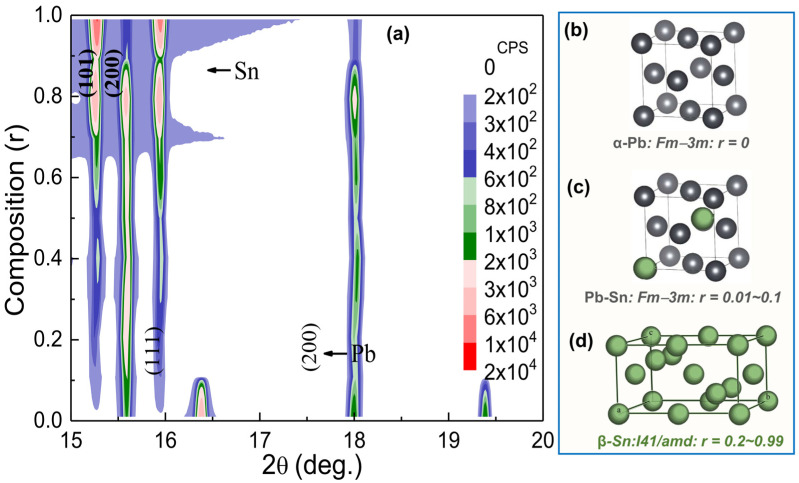
(**a**) a 2D plot of XRD patterns of Sn_r_Pb_1-r_ bimetallic nanoalloys over a narrow range of scattering angle 2θ; the crystalline structure of (**b**) α-Pb; (**c**) α-Pb/Sn solid solution; and (**d**) β-Sn, respectively.

**Figure 3:**
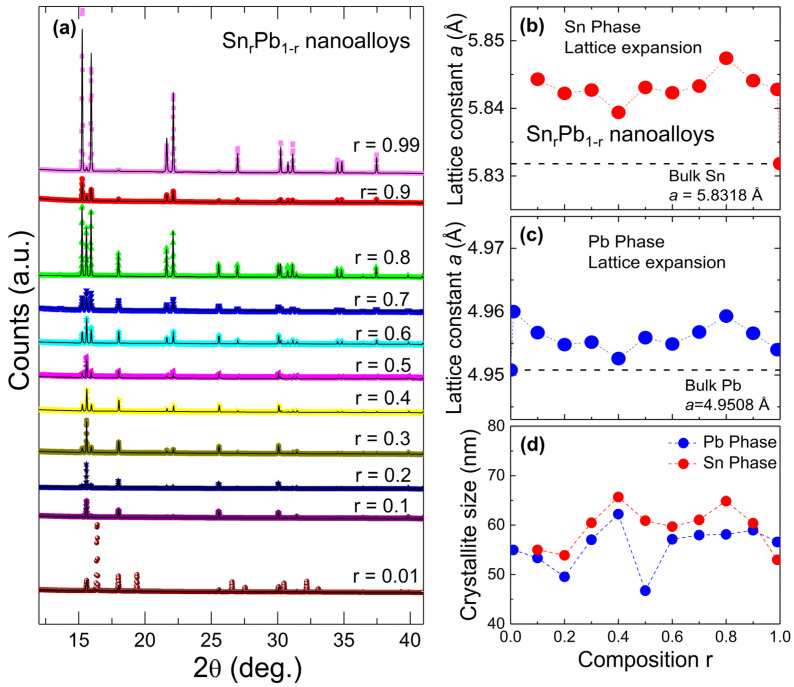
(**a**) observed (colored crosses) and Rietveld refined (solid black lines) X-ray diffraction patterns of Sn_r_Pb_1-r_ nanoalloys; (**b**,**c**) the effect of strain on the lattice constant of Pb and Sn is plotted with respect to initial composition r. Lattice expansion was observed for both Pb and Sn phases; (**d**) the variation of crystallite size with composition r for Pb and Sn phases.

A 2D plot of the X-ray diffraction pattern of Sn_r_Pb_1-r_ (0.01 ≤ r ≤ 0.99) nanoalloys over a narrow scattering range of 2θ is shown in Figure 2a, where the vertical axis represents the composition r. The “contour color fill” function provided in the Origin software was utilized to draw a 2D plot in which different colors were used to differentiate the peak intensities of the diffraction pattern. From the above 2D plot, for r = 0.01~0.1, nuclear peaks (111) and (200) indexed based on *Fm-3m* (No. 225) become visible, indicating the formation of a Pb-Sn solid solution having the same structure as that of virgin Pb, as shown in Figure 2b. Since the atomic radii of Sn (140 pm) are smaller than Pb (175 pm), Sn can occupy interstitial sites in the Pb lattices resulting in the formation of the interstitial solid solution, as shown in Figure 2c [9,10,11,12,13,14]. Sn_r_Pb_1-r_ nanoalloys that contain 0~2 at. % Sn behave like the copper-nickel alloys; a single solid solution α-Pb phase forms during solidification [12,13]. These nanoalloys are strengthened by solid-solution strengthening, strain hardening, and controlling the solidification process to refine the grain structure. A previous comprehensive study^11^ reported that the growth mechanism of SnPb solid solution is due to the grain boundary migration and sliding occurring systematically, giving rise to a series of migration markings on the surface of deformed specimens. However, for r ≥ 0.2, the Pb phase becomes unstable and shows additional diffraction picks (101) and (200) indexed based on *I4_1_/amd* (No. 141) of the Sn phase, as shown in Figure 2d. For further detailed structural analysis, Rietveld refinement of the XRD pattern of Sn_r_Pb_1-r_ bimetallic nanoalloys was carried out using the GSAS software package [15,16]. All the fitting parameters, including the lattice constant and the weight percent of constitute phases, are tabulated in Table 1. The refined patterns of the nanoalloys are shown in Figure 3a (spectra are shifted vertically for clarity). From refined XRD spectra of Sn_r_Pb_1-r_ nanoalloys, immiscible phases of Sn and Pb were observed for all compositions except the region of r = 0.01~0.1, which is in good agreement with the reported phase diagram of the Sn-Pb system [17]. From the fitted values of the lattice constant of the Pb-phase and the Sn Phase vs. r value shown in Figure 3b,c respectively, it can be observed that the Sn doped Pb (r = 0.01) results in a lattice expansion of 0.19% (Figure 3b). For the highest Sn concentration (r = 0.99), both Sn (0.19% along basal plane and *c*-axis) and Pb (0.06%) show lattice expansion (Figure 3c). The observed expansions from both Sn and Pb phases in nanoalloys could be because of the strain effect, as the thermal expansion coefficient for Sn (22 × 10^−6^ m/mK) is lower than Pb (28.9 × 10^−6^ m/mK). The crystallite size of both Sn and Pb phases were calculated using the Scherrer method: $D=\frac{k\lambda }{\beta \cos\theta }$ nm, where k is constant, θ is the angle of diffraction, λ is the incident wavelength (λ = 0.7749 Å), and β is the full width at half maximum. The obtained crystallite sizes are in nanometers as shown in Table 1, and it can be observed that the Sn-doped Pb (r = 0.01 to 0.99) results in a change in crystallite size (Figure 3b).

### 3.3. Temperature Dependence of Magnetization

**Figure 4:**
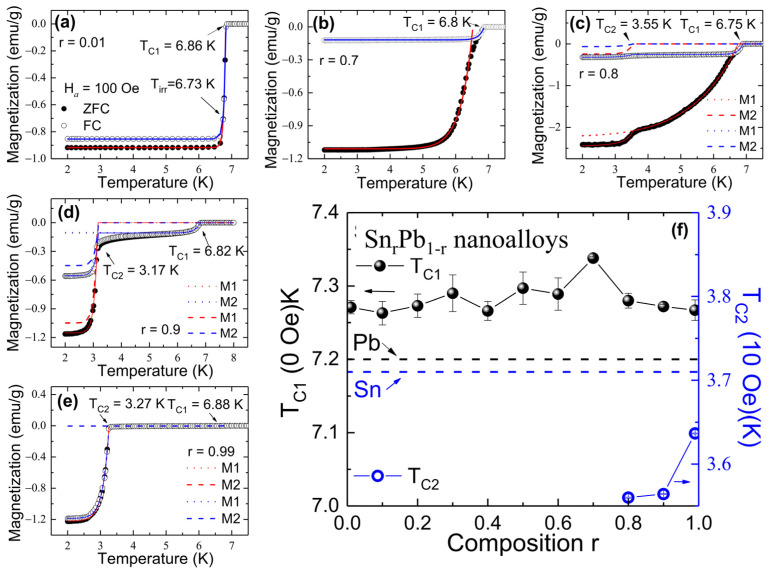
(**a**–**e**) temperature dependence of magnetization measured between 2 to 8 K in an external magnetic field of H*_a_* = 100 Oe in the ZFC and FC modes for r = 0.01, 0.7, 0.8, 0.9, and 0.99 nanoalloys. The red and blue solid curve presents the fitted curve of the modified London expression to the data; (**f**) fitted value of transition temperature T_C1_ (black sphere, see Section 3.5) and T_C2_ (blue circle, measured at 10 Oe) as a function of the initial composition. The horizontal dashed lines represent the reported values of T_C_ from bulk Pb and Sn.

Sn and Pb are weak- and strong-coupled type-I superconductors, respectively. Therefore, studying the low-temperature properties of Sn-Pb bimetallic nanoalloys will give further insight into the effect of alloying on the SC properties [17]. Furthermore, if the grain size of randomly distributed Sn and Pb in the immiscible bimetallic nanoalloys is smaller than their respective coherence length, it can further alter the SC properties due to SPE. Therefore, we have applied magnetic field and temperature-dependent magnetization measurements to study the SC properties using a magnetometer (Quantum Design MPMS VSM SQUID). A temperature dependence series of magnetization measurements between T = 2 to 8 K using an applied magnetic field of H*_a_* = 100 Oe in the ZFC and FC modes for r = 0.01, 0.7, 0.8, 0.9, and 0.99 SnPb nanoalloys are shown in Figure 4a–e, where r = 0.1~0.6 are shown in Figure S2 (Supporting Information). The observed step-like behavior of M(T) curves for r = 0.01 indicates that magnetic flux cannot penetrate the materials in a small external field of 100 Oe. However, with an increased Sn concentration, the broadening of M(T) was observed, which could be because of the inhomogeneous distribution of Pb in the Sn matrix. Interestingly, a one-step-like transition was observed from nanoalloys with initial composition r = 0.01–0.7. However, nanoalloys with r ≥ 0.8 show two-step-like transitions. The M(T) curves can be described effectively using a modified London equation with free fitting parameter p, assuming that alloying of Sn and Pb resulted in the formation of SC grains of size <*d*> obtained from the EDS mapped image of Sn_r_Pb_1-r_ nanoalloys [18,19]. The analysis of mean granular size *<d>* of segregated Sn (bright green color) and Pb (red color) elements obtained from the EDS mapping results are tabulated in Table 2. From the modified London equation, the DC magnetization can be written as:

(1) $$M\left(T\right)=a+H_{a}\times \frac{-1}{4\pi }\left\{\frac{-3}{2\rho }\left[1-6\left(\frac{\lambda_{L}}{<d>}\right)coth\left(\frac{<d>}{2\lambda_{L}}\right)+12{\left(\frac{\lambda_{L}}{<d>}\right)}^{2}\right]\right\}$$

where *ρ* is mass density which lies between the density of bulk Pb (11.35 g/cm^3^) and Sn (7.3 g/cm^3^) concerning initial composition, and *λ_L_* is the London penetration depth defined as

(2) λLT=λL01−TTC0p−1/2

where $\lambda_{L}\left(0\right)$ is a penetration depth measured at zero temperature. A good fit for the BCS predictions can be obtained using *p* = 2 for s-wave type superconductors and *p* = 3/4 for d-wave type superconductors. The power factor p defines the distribution of the transition temperature, i.e., the higher the value of *p* (≥1), the steeper the distribution of transition temperature will be. In such a scenario, the bulk-like magnetization M(T) (i.e., steep transition), can be well-fitted using *p* > 4. In these composite granular nanoalloys, the best fitting is obtained using p as a free-fitting parameter. The fitted value of p lies between 10 to 2 concerning the applied magnetic field. The obtained high value of p in a low external magnetic field is due to the flow of the shielding current around the surface of the alloy excluding the applied field detail which has been discussed in our previous work [20,21,22].

The solid line shown in Figure 4a–e is the fitted curve obtained from the modified London equation, revealing a diamagnetic Meissner state below T_C1_(100 Oe) = 6.8 (2) K (maximum, r = 0.7). At higher Sn concentration (r ≥ 0.8), a slight kink occurs in the low-temperature region at T_C2_, signaling the onset of a small superconductivity gap contributed from the Sn phase. The observed two-step behavior in r ≥ 0.8 nanoalloys can be described using a superposition of the London equation for Pb(M1) and Sn(M2), showing two transitions, T_C1_ and T_C2_. The fitted value of T_C1_(0 Oe) (for further details see Section 3.5) and T_C2_ (10 Oe) concerning initial composition r is plotted in Figure 4f. Detailed fitting parameters to M(T) curves are summarized in Table 2. The T_C1_ and T_C2_ values at 0 and 10 Oe magnetic fields are slightly higher than that of the of reported values from bulk Pb and Sn (7.2 K and 3.7 K, respectively), possibly because of the strain effect observed from the X-ray diffraction refinement [20,21].

### 3.4. Critical Fields and Theoretical Analysis

**Figure 5:**
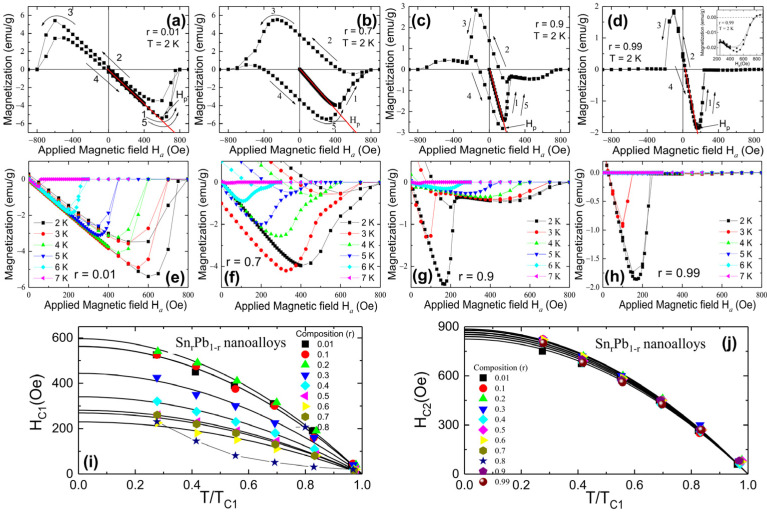
(**a**–**d**) isothermal magnetization M(H*_a_*) loops measured at 2 K for r = 0.01, 0.7, 0.9 and 0.99 nanoalloys. The solid red line is the linear fit to the first magnetization curve. Arrows mark the observed irreversible field; (**e**–**h**) the M(H*_a_*) loops measured using a protocol of H*_a_*= 0 Oe → +900 Oe → 0 Oe at higher temperature up to 7 K for r = 0.01, 0.7, 0.9, and 0.99 nanoalloys; (**i**,**j**) lower and upper critical field as a function of temperature. Solid lines fit the power law mentioned in the text.

To understand the applied magnetic effect on the superconducting properties of Sn_r_Pb_1-r_ bimetallic nanoalloys, the field dependence of magnetization M(H*_a_*) below T_C_ over ± 1200 Oe field was carried out. Figure 5a–d shows the four selected M(H*_a_*) loops measured at 2 K for r = 0.01, 0.7, 0.9, and 0.99 nanoalloys, respectively. Details of the M(H*_a_*) loops measured at 2 K for r = 0.1 to 0.6 nanoalloys are shown in Figure S3 (Supporting Information). Type-I-like M(H*_a_*) loops were observed for r = 0.01 and 0.99, whereas M(H*_a_*) loops of all remaining nanoalloys show type-II-like behavior reflecting the character of the magnetic flux penetration into the superconducting nanoalloys. The Meissner effect was observed in the low field region from M(H*_a_*). It deviated from linearity after reaching the lower critical field H_C1_, as shown in Figure 5a–d, where solid lines represent the linear fit. Above the upper critical field H_C2_ (pointed by the arrow in Figure 5a–d), magnetization M(H*_a_*) eventually turns to M = 0 states. To estimate the temperature dependency of H_C1_ and H_C2_, M(H*_a_*) loops were measured using a protocol of H*_a_*= 0 Oe → +900 Oe → 0 Oe at various temperatures, as shown in Figure 5e–h for r = 0.01, 0.7, 0.9, and 0.99 nanoalloys, respectively. Details of the M(H*_a_*) loops measured at various temperatures for r = 0.1 to 0.6 nanoalloys are shown in Figure S4 (Supporting Information). Figure 5i,j shows the T/T_C1_ dependency of H_C1_ and H_C2_, which can be described using

(3) HC1T=HC101−TTC102

and

(4) HC2T=HC201−TTC102

where H_C1_(0) and H_C2_(0) are the lower and upper critical fields at zero temperature, respectively. The fit by using the above former expression yields a maximum field of H_C1_(0) = 596(7) Oe from Sn_0.2_Pb_0.8_ nanoalloy and the minimum of H_C1_(0)~240(5) Oe from Sn_0.99_Pb_0.01_ nanoalloy. The H_C1_(0) values for other nanoalloys lie between these two critical fields and are tabulated in the supporting information of Table 2. We also note that for r = 0.8, 0.9, and 0.99 nanoalloys, due to the appearance of the second superconducting gap of Sn, it is difficult to estimate H_C1_(0) values. Contrary to H_C1_, H_C2_ for Sn_r_Pb_1-r_ nanoalloys shows typical type-II superconductor temperature dependency with linear variation near T_C_(0) and tends to saturate at low temperatures. The fit by using the above later expression yields a maximum field of H_C2_(0) = 886(16) Oe from Sn_0.2_Pb_0.08_ nanoalloy and a minimum value of H_C2_(0) = 824(8) Oe from Sn_0.01_Pb_0.99_ nanoalloy.

Using the fitted values of H_C1_(0) and H_C2_(0), in the extreme type-II limit, within the framework of Ginzburg–Landau (GL) theory [23], the GL superconducting coherence ξ_GL_ length and magnetic penetration depth λ(0) can be estimated by using $\xi_{\mathrm{GL}}\left(0\right)=\sqrt{\Phi_{0}/2{\pi H}_{C2}\left(0\right)}$ and $\lambda \left(0\right)=\kappa \left(0\right)\xi_{\mathrm{GL}}\left(0\right)$, where $\Phi_{o}=\frac{h}{2e}=2.0678\times 10^{9}\;\mathrm{Oe}$Å^2^ is the quantum flux and κ0=HC202HC10. The small value of H_C2_(0) implies a long superconducting GL coherence length ξ_GL_(0) = 632 Å, λ(0) = 541 Å (r = 0.01), and the similarly large value of H_C2_ implies a short ξ_GL_(0) = 609 Å, λ(0) = 525 Å (r = 0.2). The estimated GL parameter $\kappa \left(0\right)$ for corresponding r = 0.01 and 0.2 nanoalloys are 0.856 and 0.862, indicating type-II superconductivity. In GL theory, a superconductor is called a type-I superconductor if κ0<12 and type-II superconductor if κ0>12. When the order parameter throughout the sample is constant, the GL model reduces to the London model. Furthermore, the value of λ(0) shows increasing behavior with the increase of r, possibly due to an increase in disorder. The observed enhanced value of λ(0) = 828 Å for r = 0.99 indicates the highest degree of disorders in this series of nanoalloys. Magnetic measurement is performed with an applied magnetic field H*_a_* lower than the value of H_C1_(0), shielding current flows around the sample surface to exclude H*_a_*. In this case, penetration depth λ is obtained as the penetrating length of H*_a_* at the sample edge. However, magnetic measurements were performed with H*_a_* larger than H_C1_(0) (mixed state), and the field penetrated the sample as vortices. In this case, λ is given as a decay length of H*_a_* from the center of the vortex. In conjunction with increasing λ(0) carrier mean free path, ℓ at low-temperature T = 2 K can be estimated from GL relation

(5) 1ℓ=ξo0.8820.546ξGL201−TTC1−1ξo2

where ξ_o_ is the BCS coherence length [20,24]. The lowest value of ℓ= 1260 Å was observed for r = 0.2 nanoalloy. In addition, the calculated values of the thermodynamic critical field, $H_{\mathrm{TC}}\left(0\right)\approx \sqrt{H_{C1}\left(0\right)H_{C2}\left(0\right)},$ shows a maximum value of ~727 Oe for r = 0.2 nanoalloy. The estimated superconducting parameters for all the SnPb nanoalloys are summarized in Table 2. The observed value of ℓ is higher than that of the atomic spacing of Pb and Sn, indicating that the observed slight enhancements in the values of T_C_ could be due to the strain effect [21,22,25,26,27,28]. Furthermore, note that estimated values of ξ_GL_(0) and ℓ for both high or lower (i.e., Sn concentration) are close to the superconductor parameter of pure Pb, which could either be due to segregating of a fraction of Pb within Sn matrix or superconducting the proximity effect. Therefore, with the increase of Sn concentration, coupling strength could show decreasing behavior [29].

### 3.5. Superconducting Coupling Strength

**Figure 6:**
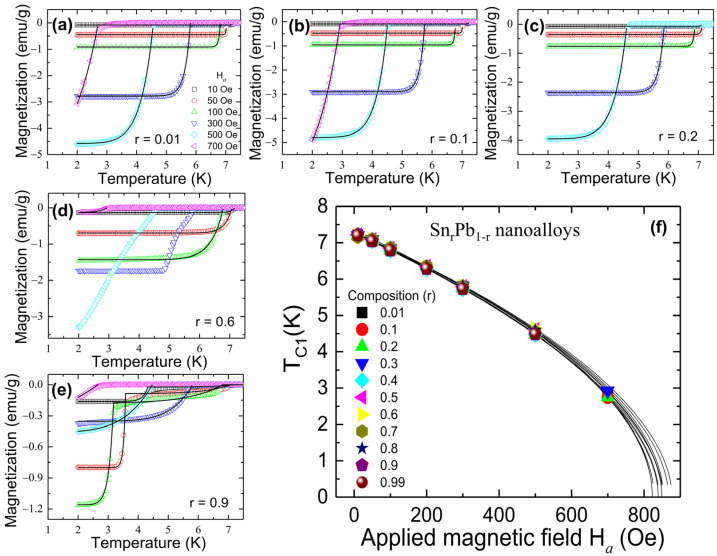
(**a**–**e**) the effect of applied field H*_a_* on the transition temperature T_C1_ of Sn_r_Pb_1-r_ nanoalloys; (**f**) the H*_a_* dependency of T_C1_. The solid line is fitted to the expression given in the text.

**Figure 7:**
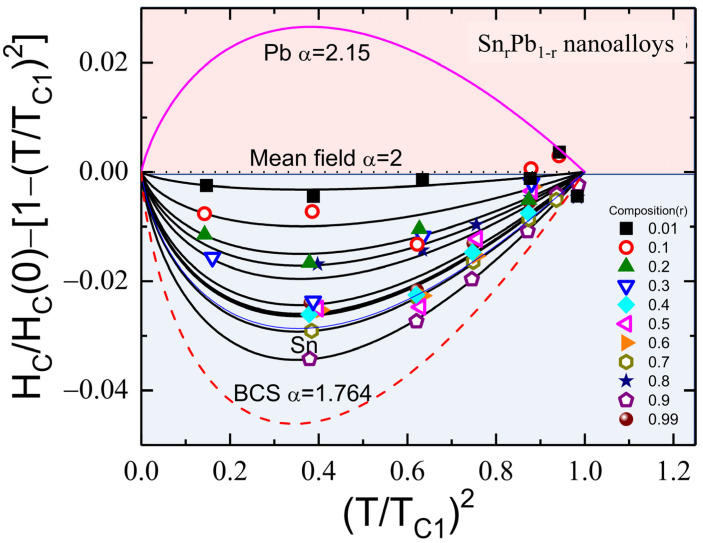
Deviation of H_C_(T)/H_C_(0) from the parabolic dependence of 1 − (T/T_C1_)^2^ fitted to the expression H_C_(T)/H_C_(0) = [1 − (T/T_C1_)^α^], where α represents the coupling strength of the superconductor.

For further detailed analysis, field-dependent ZFC M(T) curves were measured with different external magnetic fields H*_a_* varying from 10 to 700 Oe and fitted with the modified London equation as shown in Figure 6a–e. More details of the M(T) measured at various applied magnetic fields for r = 0.1 to 0.6 nanoalloys are shown in Figure S5 (Supporting Information). The H*_a_* dependency of T_C1_ shown in Figure 6f can be fitted using $T_{C1}\left(H_{a}\right)=T_{C1}\left(0\right){\left[1-H_{a}/H_{C}\left(0\right)\right]}^{\gamma }$, where γ is a fitting parameter. The obtained highest critical field H_C_(0) at T = 0 K, zero-field critical temperature T_C1_(0), and the fitting parameter γ are tabulated in Table 3. The fitted value of H_C_(0) represents the upper critical field, and its value matches very well with the fitted value of H_C2_(0) (Equation (4); Table 2). The relative coupling strength of a superconducting system can be revealed in the deviation of H_C_(T)/H_C_(0) from the parabolic dependence of $1-{\left(T/T_{C1}\right)}^{2}$ [30]. It is known that weakly coupled systems yield negative deviations, while strongly coupled systems yield positive deviations (marled as red color) [31]. A negative deviation (marked as blue color) was observed for all Sn_r_Pb_1-r_ nanoalloys, as shown in Figure 7, which can be described using the α-model defined as $\frac{H_{C}\left(T\right)}{H_{C}\left(0\right)}=\left[1-{\left(\frac{T}{T_{C1}}\right)}^{\alpha }\right]$, where the fitting parameter $\alpha =\Delta_{o}/k_{B}T_{C1}$ represents the coupling strength (summarized in Table 3). From Figure 7, we note that coupling strength α subverts with an increase of Sn concentration and shows a minimum of α = 1.821(3), T_C1_(0) = 7.272(3) K for r = 0.9 nanoalloy, which is more vital than BCS coupling strength of 1.747 [32,33]. The observed subverted coupling strength as compared to strong-coupled Pb is possibly due to the propagation of SPE through the weak-coupled Sn matrix. The observed results suggest that the coupling strength of strong-coupled Pb can be tuned through SPE in Sn_r_Pb_1-r_ bimetallic nanoalloys. A slight enhancement of superconducting transition temperature (T_C_) was observed from the magnetization, revealing a main diamagnetic Meissner state below T_C_ = 7.338(2) K and a critical field of H_C_ = 852(5) Oe from Sn_0.7_Pb_0.3_ mixed-phase nanoalloy associated with strain effect.

**Figure 8:**
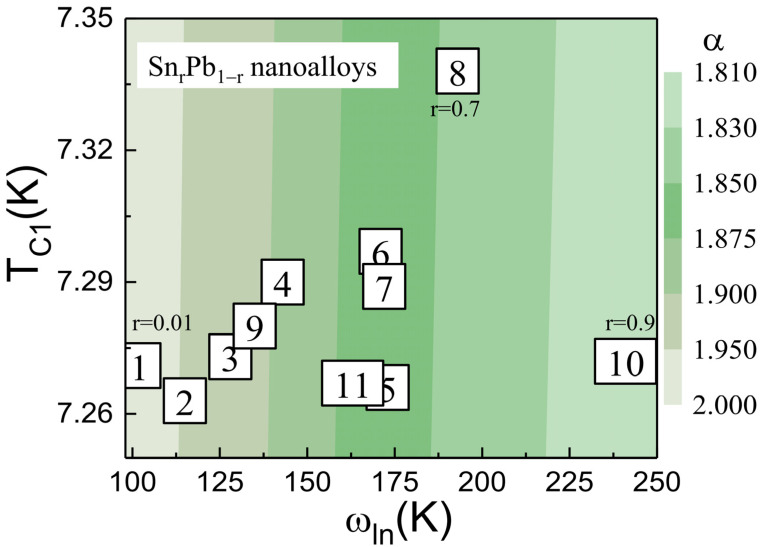
A 2D plot of observed T_C_ versus estimated ω_ln_ from the equation explained in the text where the various green colors represent weak-coupled strength α, respectively.

To investigate the effect of coupling strength on average logarithmic phonon energy ω_ln_ and the electron–phonon coupling constant *λ_ep_*, we utilized the Eliashberg theory-based McMillian formulation [34]. The corrections of the BCS values by strong electron–phonon interactions have been deduced in the following approximate analytic formulas that link ω_ln_/T_C1_(0) to experimental thermodynamic quantities:

(6) $$\alpha =\frac{\Delta \left(0\right)}{k_{B}T_{C1}\left(0\right)}=1.764\left[1+12.5{\left(T_{C1}\left(0\right)/\omega_{\ln}\right)}^{2}\times \ln\left(\omega_{\ln}/2T_{C1}\left(0\right)\right)\right]$$

where ∆(0) is a superconducting energy gap [35]. Figure 8 displays a 2D plot of observed T_C_ versus estimated ω_ln_ from Equation (6), where the green colors represent weak-coupled strength α. The value of ω_ln_ is helpful for the description of the superconducting properties of conventional superconductors. The most weak-coupled Sn_0.9_Pb_0.1_ nanoalloy (sample #10) has α = 1.821(3) and T_C1_(0) = 7.272(3) K and shows the maximum phonon energy of ω_ln_ = 241 K (phonon hardening). An electron–phonon coupling constant *λ_ep_* can be estimated from the McMillan equation,

(7) $$\frac{T_{C1}\left(0\right)}{\omega_{\ln}}=\frac{1}{1.2}exp\left[\frac{-1.04\left(1+\lambda_{ep}\right)}{\lambda_{ep}-\mu^{*}\left(1+0.62\lambda_{ep}\right)}\right]$$

where μ* is the Coulomb pseudopotential described the Coulomb pseudopotential to represent the repulsive part of the pairing interaction and to estimate *λ_ep_*; we set μ* = 0.11 [34]. The obtained values of *λ_ep_* are tabulated in Table 3. From calculated values of $\omega_{\ln}$ and *λ_ep_*_,_ it becomes clear that T_C1_ is a combined effect of both electron–phonon coupling strength and phonon energy. The minimum *λ_ep_* = 0.685 and maximum ω_ln_ = 241 K were observed for Sn_0.9_Pb_0.1_ bimetallic nanoalloy. The above value of *λ_ep_* is smaller than that of pure Pb and Sn, *λ_ep_* = 1.580 and 0.761, respectively. From the above finding, it is clear that the propagation of superconductivity through SPE weakened the electron–phonon coupling strength and hardened the phonon of the Pb phase.

## 4. Discussions

**Figure 9:**
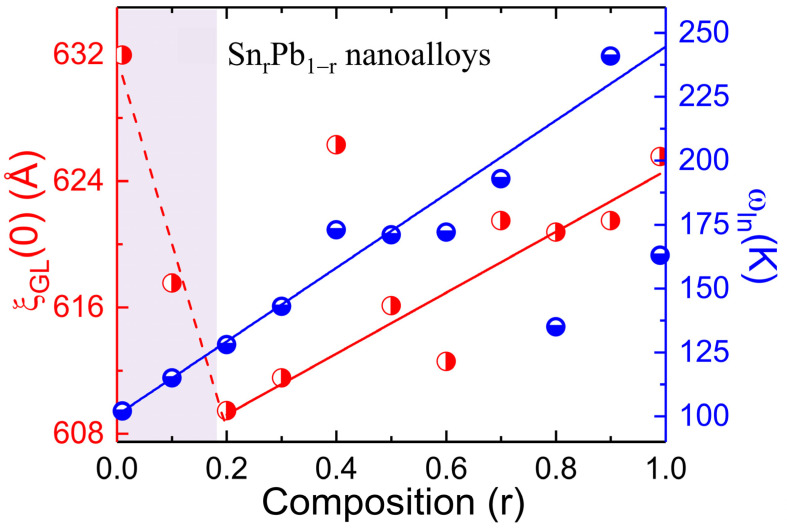
The plot of composition r depends on the GL coherence length and the phonon energy. The red dashed line and solid blue lines are guides for the eyes.

According to Pippard, Cooper pair size manifests as a superconducting energy gap ∆(0), which becomes shorter with increased coupling strength [36]. On the other hand, phonon-mediated superconductors subversion of coupling strength was observed due to the phonon hardening effect. Therefore, the estimated values of GL coherence length ξ_GL_(0) and the average logarithmic phonon energy ω_ln_ are linked with the coupling strength of the superconductor. Figure 9 depicts the initial composition r dependence of calculated values of ξ_GL_(0) and ω_ln_ from Sn_r_Pb_1-r_ (0.01 ≤ r ≤ 0.99) bimetallic nanoalloys. For an initial Sn concentration from r = 0.01 to 0.2, a steep drop behavior of ξ_GL_(0) was observed (the dashed red line is guided for the eyes), while the two elements of Pb and Sn form a solid solution of the α-Pb phase. The resultant phase diagram is thus completely different from those of other composition regions r = 0.2~0.99, typical immiscible bimetallic superconductors of Sn and Pb compounds, in which ξ_GL_(0) and ω_ln_ increase when Sn is substituted for Pb in the strong-coupled superconducting “parent” compounds. Notably, the solid solutions in the superconducting range exhibit ξ_GL_(0) anomalies at r = 0.01–0.1. The behavior is reminiscent of the disorder, and substitution in weak coupling superconductivity could enhance the electron–phonon coupling constant. A simplified physical model of electron–phonon coupling has been developed by Gao et al. to allow heat transfer from phonons to electrons and applied to study defects or disorders as a function of the strength of electron–phonon coupling [37]. The number of point defects produced in the primary damage state increases with the strength of electron–phonon coupling, signaling that the defect or disorder/substitution plays a role in the increasing strength of the electron–phonon coupling constant. A maximum of *λ_ep_* = 1.046 from r = 0.01 and *λ_ep_* = 0.97 from r = 0.1 (as shown in Table 3) indicates that the similarity suggests the enhancement of the electron–phonon coupling in the present case also has a substitutional origin. On the other hand, ω_ln_ shows a linear increasing behavior (the solid blue line is guided for eyes) with the decrease of coupling strength from r = 0.01 to 0.9, above which fluctuations were observed. The observed results agree with theoretical findings except for the high Pb concentration (r = 0.01 and 0.1) region.

## 5. Conclusions

Sn_r_Pb_1-r_ (0.01 ≤ r ≤ 0.99) bimetallic nanoalloys were prepared using the simple physical annealing system. The observed discrepancy between the estimated atomic % using EDS and initial composition could be because of segregated grains of Sn and Pb. EDS-mapped images show the inhomogeneous distribution of nano to micrometer size Sn and Pb segregated grains. SR-XRD refinement carried out using Rietveld analysis reveals the formation of the pure Pb-solid solution and immiscible mixed-phase nanoalloys. The effect of strain was observed on the lattice constants of Sn and Pb phases in all immiscible nanoalloys. Magnetization measurements revealed slight enhancement in the superconducting transition T_C_ for strained nanoalloy when fitted to modify the London equation. The coupling strength *α*, electron–phonon coupling constant *λ_ep_*, and average logarithmic phonon energy ω_ln_ were estimated for all nanoalloys using the α-model and McMillian formulation. The estimate α, *λ_ep_* showed subverted behavior and phonon hardening due to the propagation of SPE through the Sn matrix. The estimated sizes of the Sn phase increased from 50~60 nm as the composition r increased from 0.2~0.9, reflecting the excess surface contributions of the Sn matrix giving rise to enhanced propagation of SPE. In the immiscible regime, the behavior of ξ_GL_(0) is the same as that of the phonon energy, ω_ln,_ i.e., both physical parameters increase with the decrease of coupling strength α, revealing a superconducting proximity effect between immiscible Sn and Pb granules in the range of r = 0.2~0.9.

## Figures and Tables

**Table 1:** Summary of best fitted Rietveld refined fitting parameters of the Sn and Pb phases from the Sn_r_Pb_1-r_ nanoalloys using the GSAS software package: lattice constant (*a*, *b*, *c*), weighted residue (Rwp), residual of least square refinement (Rp), a squared ratio between Rwp and Rexp (χ^2^), crystalline size, and weight fractions (Wt. fraction).

Sn_r_Pb_1-r_	Lattice Constants (Å)			Rwp	Rp	χ^2^	Crystalline Size (nm)		Wt. Fraction (%)	
	Pb Phase	β-Sn Phase					Pb Phase	Sn Phase		
	*a* = *b* = *c*	*a* = *b*	*c*						Pb	β-Sn
0.01	4.9600 (1)			0.3554	0.1881	21.45	55		100	0
0.1	4.9567 (1)	5.8443 (4)	3.1884 (3)	0.0265	0.0111	0.1184	53	55	75.123	24.877
0.2	4.9548 (1)	5.8422 (3)	3.1859 (2)	0.0424	0.0215	0.2699	50	54	69.128	30.872
0.3	4.9552(1)	5.8427 (1)	3.1875 (1)	0.031	0.017	0.1587	57	60	68.782	31.218
0.4	4.9526 (1)	5.8394 (1)	3.1855 (1)	0.0344	0.018	0.1888	62	66	65.555	34.445
0.5	4.9559 (1)	5.8431 (2)	3.1877 (1)	0.0442	0.0229	0.2843	47	61	50.246	49.754
0.6	4.9549 (1)	5.8423 (1)	3.1872 (1)	0.0555	0.0306	0.4864	57	60	36.476	63.524
0.7	4.9568 (1)	5.8433 (1)	3.1894 (1)	0.0459	0.0246	0.3812	58	61	54.848	45.152
0.8	4.9593 (1)	5.8474 (1)	3.1894 (1)	0.0716	0.04	1.175	58	65	41.589	58.411
0.9	4.9566 (2)	5.8441 (1)	3.1877 (1)	0.0479	0.0236	0.3648	59	60	34.294	65.706
0.99	4.9540 (9)	5.8428 (2)	3.1878 (1)	0.1424	0.0903	5.461	57	53	0.775	99.225

**Table 2:** Summary of obtained physical parameters: mean granular size <*d*>, critical field H_C1_(0), upper critical field H_C2_(0), and thermodynamical critical field H_C_(0), Ginzburg–Landau coherence length ξ_GL_(0), Ginzburg–Landau parameter k, penetration depth λ(0), and mean free path ℓ of Sn_r_Pb_1-r_ nanoalloys estimated within the superconducting state.

Sn_r_Pb_1-r_	<*d*> nm	H_C1_(0) Oe	H_C2_(0) (Oe)	H_TC_(0) (Oe)	ξ(0) (Å)	λ (Å)	ξ_GL_(0) (Å)	$\kappa \left(0\right)$	$\ell \left(Å\right)$
0.01	121 (10)	562 (7)	824 (8)	681	896	541	632	0.856	1536
0.1	105 (9)	563 (8)	863 (13)	697	897	540	618	0.875	1340
0.2	80 (5)	596 (7)	886 (16)	727	896	525	609	0.862	1260
0.3	79 (4)	444 (10)	880 (12)	625	894	609	612	0.995	1298
0.4	77 (5)	340 (4)	839 (10)	534	897	696	626	1.111	1452
0.5	70 (3)	281 (3)	867 (17)	494	893	765	616	1.242	1358
0.6	81 (4)	229 (7)	877 (13)	448	894	848	613	1.384	1309
0.7	75 (5)	269 (5)	852 (13)	479	888	782	622	1.258	1472
0.8	75 (4)	260	854 (10)	471	895	795	621	1.281	1397
0.9	69 (3)	250	852 (11)	462	896	811	622	1.305	1398
0.99	46 (3)	240	841 (13)	449	897	828	626	1.323	1444

**Table 3:** Summary of obtained physical parameters, such as critical temperature T_C1_(0), critical field H_C_(0), fitting parameters γ, coupling strength α, phonon energy ω_ln_, and electron–phonon coupling constant λ_ep_ of Sn_r_Pb_1-r_ nanoalloys, estimated within the superconducting state.

Sample#	Sn_r_Pb_1-r_	T_C1_(0) (K)	H_C_ (Oe)	γ	α	ω_ln_ (K)	*λ_ep_*
1	0.01	7.271 (9)	823 (5)	0.505 (8)	1.983 (10)	102	1.046
2	0.1	7.263 (16)	823 (8)	0.513 (13)	1.947 (15)	115	0.97
3	0.2	7.273 (16)	828 (8)	0.521 (14)	1.920 (16)	128	0.913
4	0.3	7.290 (25)	850 (15)	0.528 (23)	1.896 (23)	143	0.862
5	0.4	7.266 (13)	840 (31)	0.537 (31)	1.861 (12)	173	0.785
6	0.5	7.297 (22)	868 (59)	0.537 (57)	1.864 (19)	171	0.79
7	0.6	7.289 (22)	878 (64)	0.537 (60)	1.863 (20)	172	0.788
8	0.7	7.338 (2)	852 (5)	0.541 (5)	1.847 (1)	193	0.751
9	0.8	7.280 (10)	854 (27)	0.524 (26)	1.909 (10)	135	0.887
10	0.9	7.272 (3)	853 (8)	0.549 (8)	1.821 (3)	241	0.685
11	0.99	7.267 (14)	841 (33)	0.534 (33)	1.872 (12)	163	0.807

## Data Availability

Not applicable.

## Supplementary Materials

The following supporting information can be downloaded at: https://www.mdpi.com/article/10.3390/nano12234323/s1, Figure S1: The EDS mapped SEM images; Figure S2: Temperature dependence of magnetization; Figure S3: The isothermal magnetization M(H*_a_*) loops; Figure S4: The M(H*_a_*) loops measured using protocol 0 Oe → +900 Oe → 0 Oe field; Figure S5: Temperature dependence of ZFC magnetization of Sn_r_Pb_1-r_ bimetallic nanoalloys.
