# Supplementary material for: Structural and Superconducting Proximity Effect of SnPb Bimetallic Nanoalloys
Source: Nanomaterials (Basel). 2022 Dec 5;12(23):4323. doi: 10.3390/nano12234323 (PMC9738066; doi:10.3390/nano12234323)

Supplementary information

Structural and Superconducting Proximity Effect of SnPb Bimetallic Alloys

Ashish Chhaganlal Gandhi,[a,b] K. Manjunatha,[a] Ting-Shan Chan,[b] and Sheng Yun Wu[a,*]

[a] Department of Physics, National Dong Hwa University, Hualien 97401, Taiwan

[b] Department of Electrical Engineering, National Tsing Hua University, Hsinchu 30013, Taiwan

[c] National Synchrotron Radiation Research Center, Hsinchu 30076, Taiwan

*Corresponding author: sywu@mail.ndhu.edu.tw

Figure S1 (a)-(k) The EDS mapped SEM images of Sn_rPb_{1-r} bimetallic nanoalloys, where red and green represent the atomic percent of Pb and Sn, respectively. The EDS mapping images of Sn_rPb_{1-r} nanoalloy show the distribution of segregated Sn (bright green color) and Pb (red color) elements with grain size varying from <d>=121(10) ~ 46(3) nm.

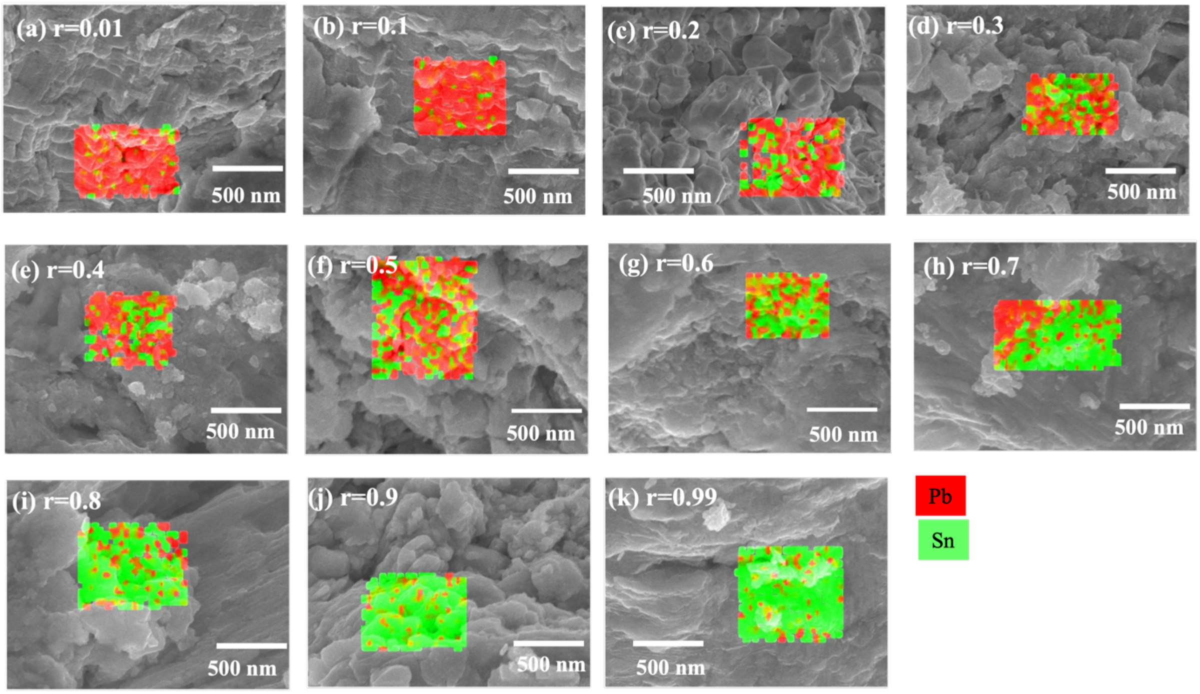

Figure S2 (a)–(f) Temperature dependence of magnetization measured between 2 to 8 K in an external magnetic field of H_a = 100 Oe in the ZFC and FC modes for r = 0.1, 0.2, 0.3, 0.4, 0.5 and 0.6 nanoalloys. The red and blue solid curve presents the fitted curve of the modified London expression to the data.

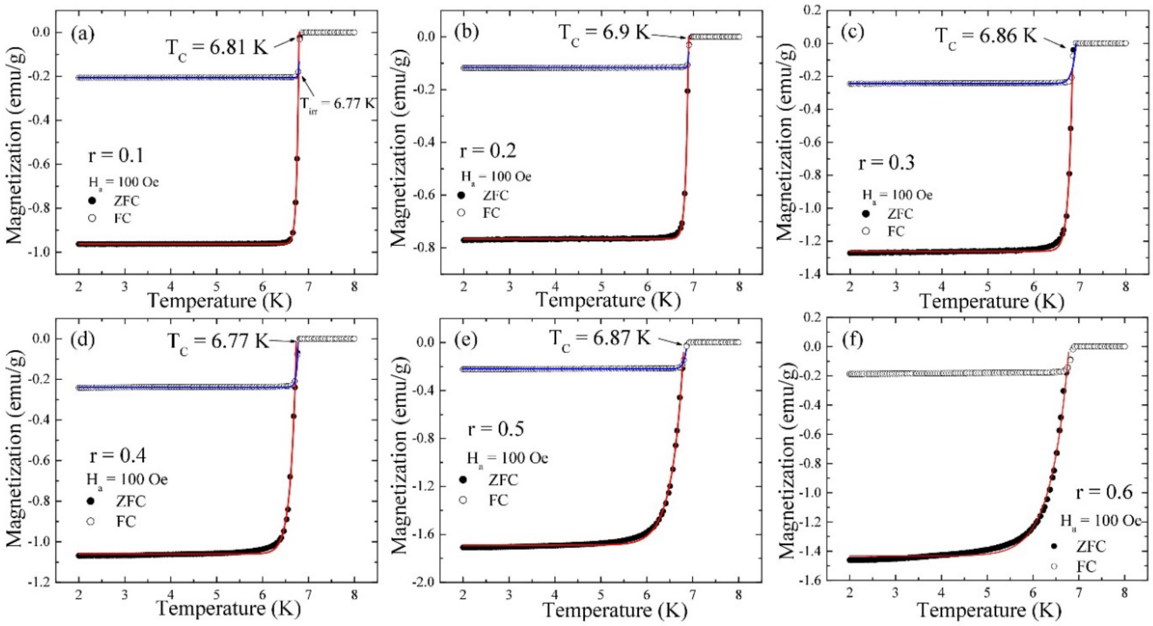

Figure S3 (a)-(f) The isothermal magnetization M(H_a) loops measured at 2 K for r = 0.1-0.6 nanoalloys.

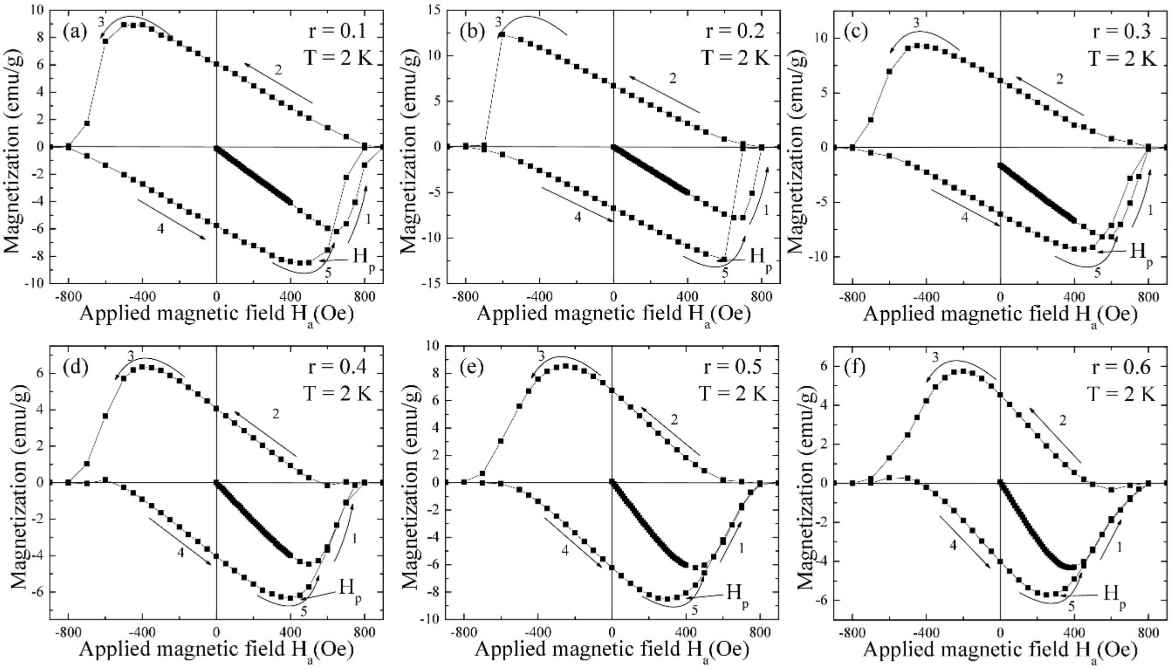

Figure S4 (a)-(f) The M(H$_a$) loops were measured using protocol 0 Oe → +900 Oe → 0 Oe field at a higher temperature up to 7 K for r = 0.1-0.6 nanoalloys.

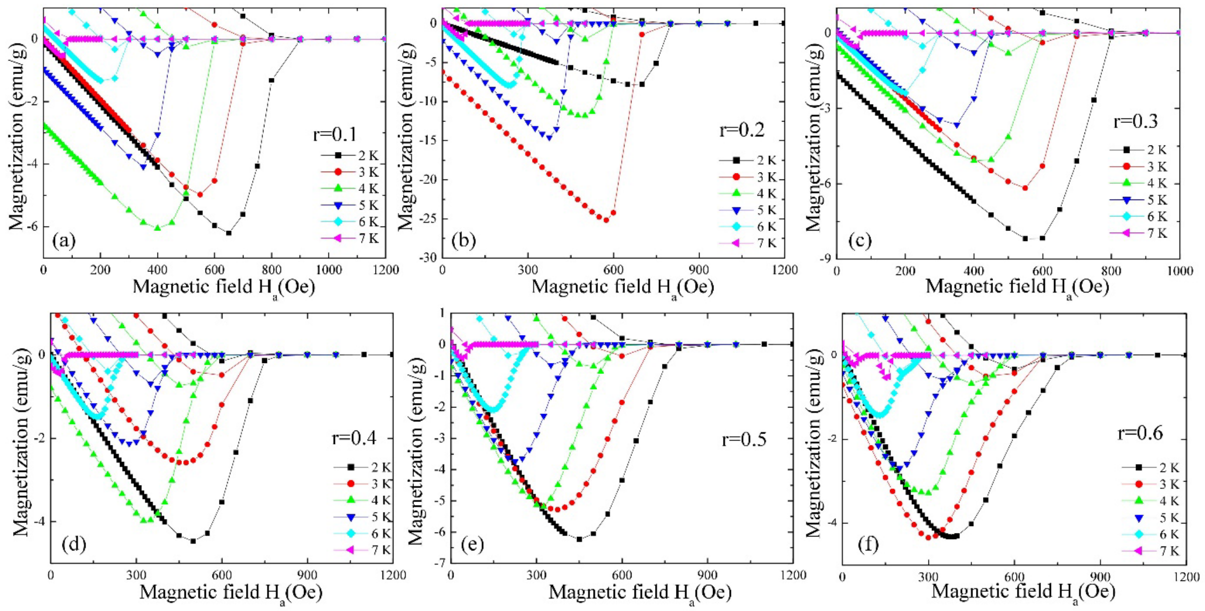

Figure S5 (a)-(k) Temperature dependence of ZFC magnetization M(T) measured between 2 to 8 K with different external magnetic fields varying from 10 to 700 Oe for all Sn_rPb_{1-r} bimetallic nanoalloys. The solid curve presents the fitted curve of the modified London expression to the data.

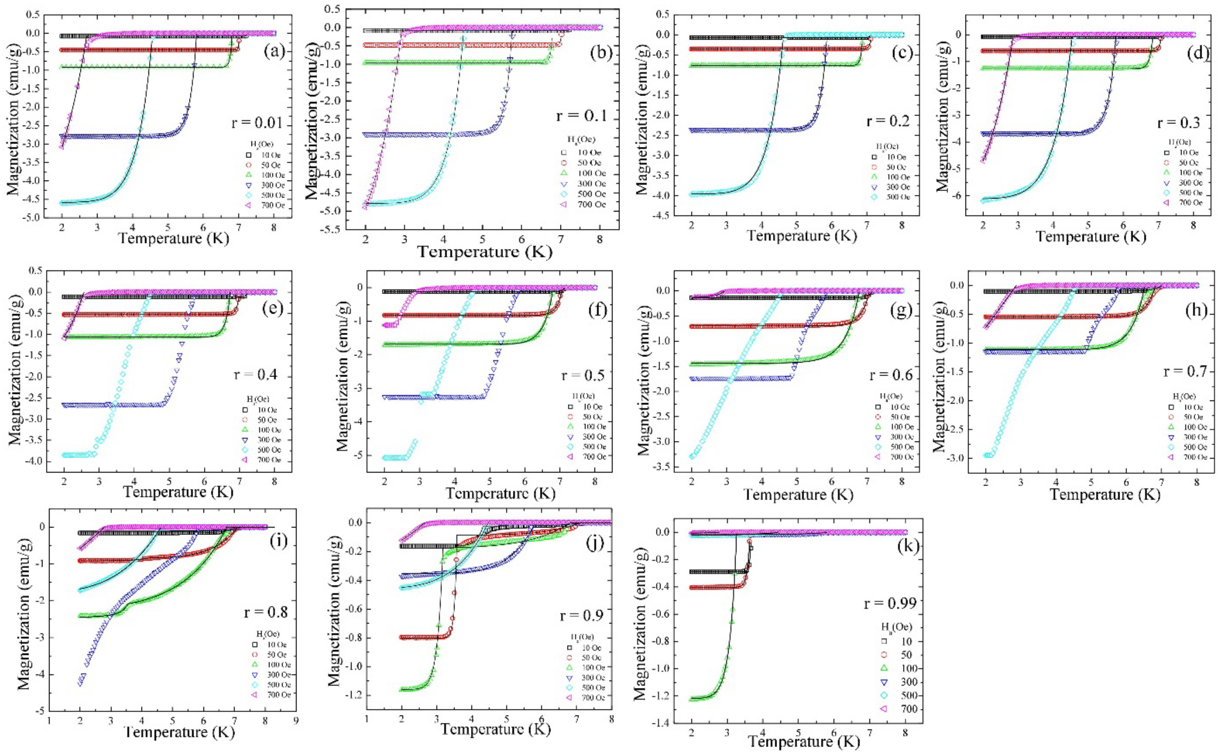

Supplement: Supplementary file 1 [file nanomaterials-12-04323-s001.zip › nanomaterials-2074043-SI.pdf]
